# Supplementary material for: A Functional Single-Nucleotide Polymorphism Upstream of the Collagen Type III Gene Is Associated with Catastrophic Fracture Risk in Thoroughbred Horses
Source: Animals (Basel). 2023 Dec 28;14(1):116. doi: 10.3390/ani14010116 (PMC10778232; doi:10.3390/ani14010116)
Supplement: Supplementary file 1 [file animals-14-00116-s001.zip › animals-2750382-supplementary.pdf]

Supplemental Material

Table S1. List of primer sequences used in qPCR.

Gene	Forward primer	Reverse primer
18S RNA	CCCAGTGAGAATGCCCTCTA	TGGCTGAGCAAGGTGTTATG
GAPDH	CGACCACTTTGTCAAGCTCA	GTCCACCACCCTATTGCTGT
ACTB	CCAGCACGATGAAGATCAAG	GTGGACAATGAGGCCAGAAT
COL1A1	TGCGAAGACACCAAGAACTG	GACTCCTGTGGTTTGGTCGT
SPARC	TGGCGAGTTTGAGAAGGTGT	TTTGCAAGGCCCGATGTAGT
SPP1	AGCCCCAGGAAAAATCGCTG	GGCATAAGCAAATCACGGCA
IBSP	GGACTGCACACGGAAACAATC	ACAGGCCATTCCCAAAATGC
RUNX2	CCAAGTGGCAAGGTTCAACG	AACTCTTGCCTCGTCCACTC
BGALP	GTCTCGGGGTTCCAAGGTTA	AATCTCTGGTAGCTGTGTTGGT
ITGAV	GATGCTGATGGACAGGGATT	AAACTACCAGGACCACCAAGAA
CALCRL	TTTGTGTTTCTCTTGCCTTTTT	TCTTCTGATTCTGCTGTGACAA
GULP1	GCCCAAAGGAACAGAAGTTG	GGAATTTTCTGGCCTTCAGA
SLC401A	GCCAACTACCTGACCTCTGC	AAAGTGCCACATCCGATCTC
MSTN	TGACAGCAGTGATGGCTCTT	TTGGGTTTTCCTTCCACTTG
C2orf88	CCTGTCCCAGGAAATCTTGA	GATGTTATTGCCTGCCCACT
STAT1	TCTGCAGCTGTCTGAAGGAG	TGAATATTCCCCGACTGAGC
GLS	CCTAGCTTGGAAGATTTGCTG	CAGACGTTCGCAATCCTGTA
ZNF804A	ATGACCATGCTCACAAGCAG	TTTCGAGCAAATTCCCTTTG
COL5A2	AGGAGAGAGAGGCCCAAAAG	CTCCATCAATTCCCTGAGGA
COL3A1	CTGGTGCTAATGGTGCTCCT	TCTCCTTTGGCACCATTCTT
SOX11	GTTCATGGTGTGGTCCAAGA	GCTGTCCTTCAGCATTTTCC

Table S2. Antibodies used in immunocytochemistry.

Antibody	Company and Catalogue number	Dilution
Rabbit anti-SOX11	Sigma; sab4200450	1:200
Rabbit anti-COL3A1	Abcam; ab7778	1:100
Rabbit anti-RUNX2	Santa-Cruz; sc10758	1:50
Rabbit anti-STAT1	Abcam; ab109320	1:200
Goat anti-rabbit alexafluor 594	ThermoFisher; A11012	1:200

Table S3. Minor allele frequencies of the SNP in different breeds.

Breed	Number of horses	Minor Allele Frequency (MAF)	Reference
Arabian	38	0.00	Durward-Akhurst et al 2021 [53]
Belgian	20	0.00	Durward-Akhurst et al 2021 [53]
Clydesdale	19	0.00	Durward-Akhurst et al 2021 [53]
Franches Montagne	30	0.13	Durward-Akhurst et al 2021 [53]
Icelandic	17	0.00	Durward-Akhurst et al 2021 [53]
Jeju pony	21	0.02	Durward-Akhurst et al 2021 [53]
Morgan	22	0.11	Durward-Akhurst et al 2021 [53]
Other (36 breeds)	127	0.05	Durward-Akhurst et al 2021 [53]
Quarter horse	103	0.66	Durward-Akhurst et al 2021 [53]
Shetland	55	0.00	Durward-Akhurst et al 2021 [53]
Standardbred	59	0.04	Durward-Akhurst et al 2021 [53]
Thoroughbred (US and European)	76	0.53	Durward-Akhurst et al 2021 [53]
Welsh pony	20	0.00	Durward-Akhurst et al 2021 [53]
>25 different breeds	88	0.13	Jagannathan et al 2019 [51]
Thoroughbred (Japanese)	101	0.50	Tozaki et al 2021 [52]
UK Thoroughbred fracture cases	91	0.32	N/A
UK Thoroughbred fracture controls	86	0.22	N/A

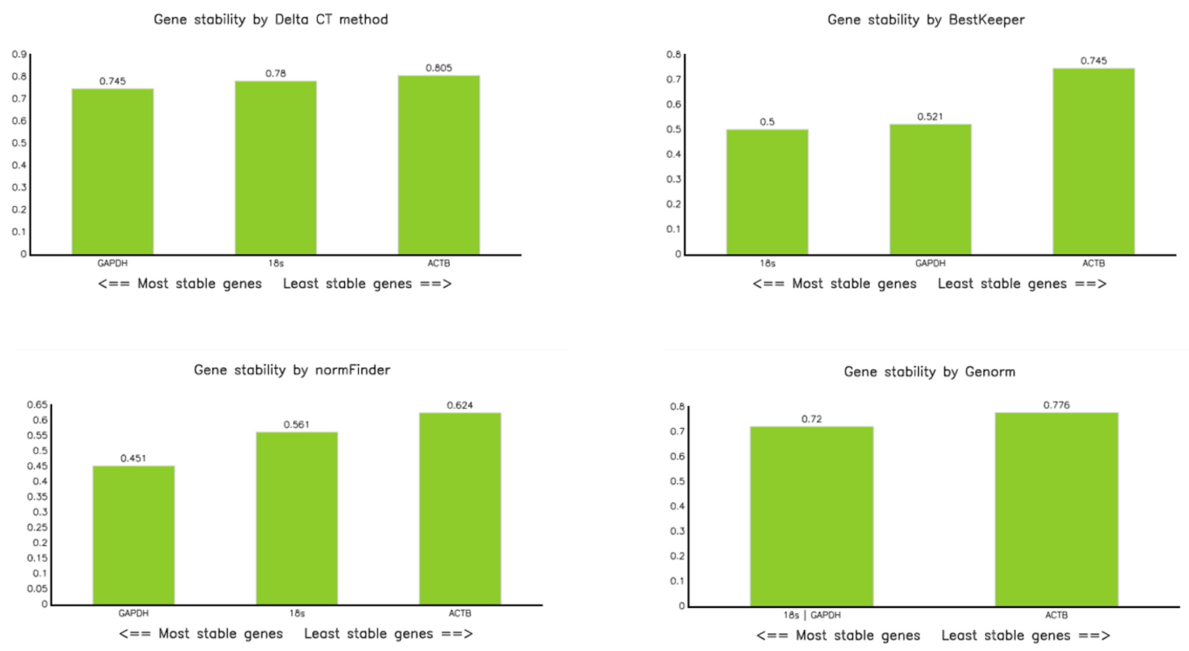

Figure S1. Output from RefFinder showing the stability testing of three housekeeping genes. This demonstrated that 18S RNA and GAPDH were more stable than ACTB across four different housekeeping computational tools.

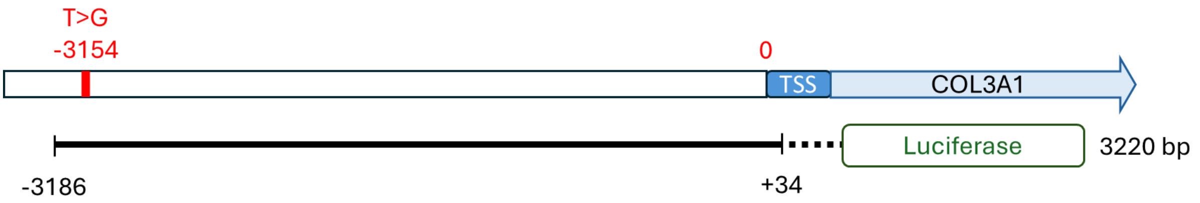

Figure S2. Diagram of the luciferase assay construct relative to the COL3A1 gene transcriptional start site (TSS). The positions in base pairs are indicated relative to the TSS (position 0). The position of the SNP (T>G) is also highlighted.

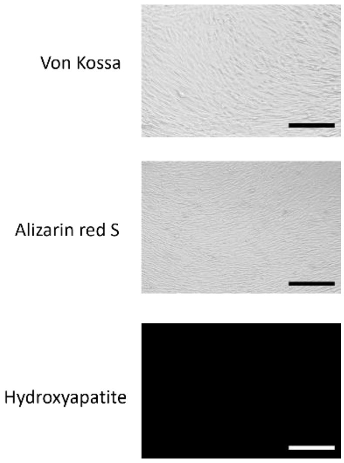

Figure S3. Undifferentiated skin fibroblasts cultured under normal conditions (without osteogenic factors) show no positive staining to indicate matrix mineralisation.

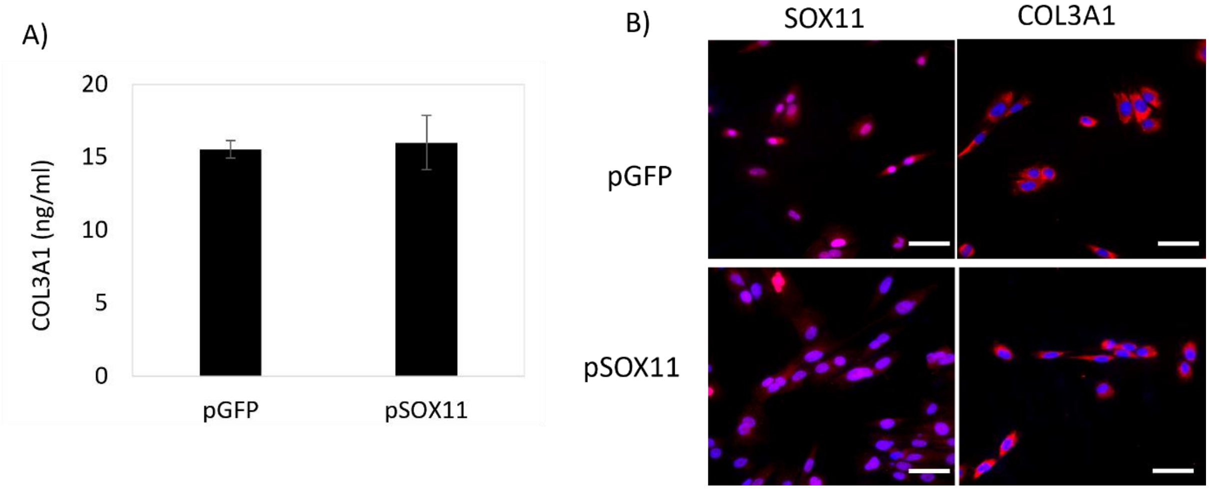

Figure S4. COL3A1 protein levels do not change in response to SOX11 overexpression. A) No difference in total COL3A1 protein levels is detected using ELISA. Error bars represent the s.e.m from cells derived from three different donors. *p<0.05. B) SOX11 and COL3A1 (both shown in red) protein expression in control (pGFP) and SOX11 overexpressing (pSOX11) cells. DAPI staining of the nucleus is shown in blue. Scale bar = 40 µm. Images are representative of replicates using cells derived from three different donors.

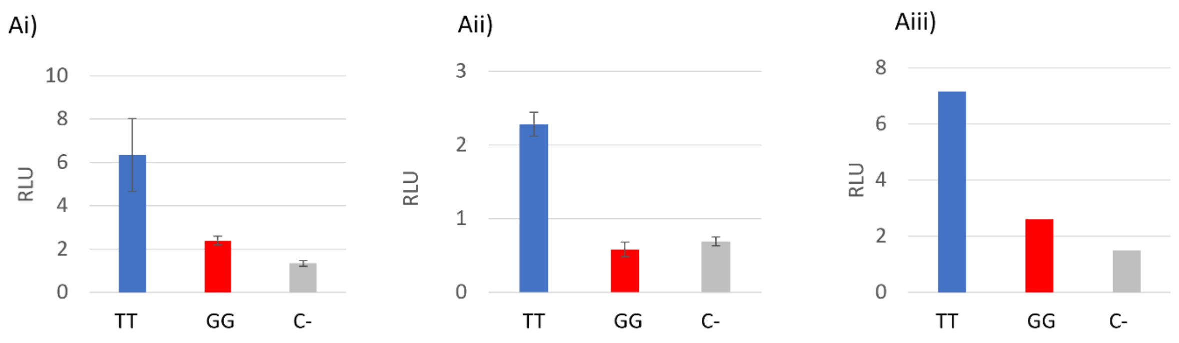

Figure S5. Luciferase assays showing the relative light unit (RLU) for the 3220 bp region upstream of COL3A1 containing either the reference allele (TT) or the alternative allele (GG) compared to a promoterless control (C-). Ai – iii) show the results of independent luciferase assays set up on different days using skin cells derived from the same horse. Error bars represent the s.e.m of three to six technical replicates.
